# Incidence of Birth Asphyxia as Seen in Central Hospital and GN Children’s Clinic both in Warri Niger Delta of Nigeria: An Eight Year Retrospective Review

**DOI:** 10.5539/gjhs.v4n5p140

**Published:** 2012-08-09

**Authors:** G. I. Mcgil Ugwu, H. O. Abedi, E. N. Ugwu

**Affiliations:** 1 Department of Paediatrics, Delta State University Teaching Hospital, Oghara, Nigeria; 2 Department of Obstetrics and Gynaecology, Delta State University Teaching Hospital, Oghara, Nigeria; 3 Chevron Hospital Warri, Nigeria

**Keywords:** birth asphyxia, warri, Nigeria

## Abstract

**Background:**

Birth asphyxia is one of the commonest causes of neonatal morbidity and mortality in developing countries. Together with prematurity and neonatal sepsis, they account for over 80% of neonatal deaths.

**Aim:**

To determine the incidence and mortality rate of birth asphyxia in Warri Niger Delta of Nigeria.

**Materials and Method:**

Recovery of case notes of all the newborn babies seen from January 2000 to December 2007 at Central Hospital Warri and GN children’s Clinic, Warri, was undertaken. They were analyzed and those with birth asphyxia were further analyzed, noting the causes, severity of asphyxia, sex of the babies, management given.

**Results:**

A total of 864 out of 26,000 neonates seen within this period had birth asphyxia. 525 (28/1000 live births) had mild asphyxia while 32% were severely asphyxiated. 61.5% of the asphyxiated were born at maternities, churches or delivered by traditional birth attendants or at home. Prolonged labour was the commonest cause of asphyxia and asphyxia was more in neonates from unbooked patients.

**Conclusion:**

The incidence of bith asphyxia in Warri is 28/1000. Majority of patients are from prolonged labour and delivery at unrecognized centres. Health education will dratically reduce the burden of asphyxia neanatorum as unsubtanciated religous beliefs have done a great havoc.

## 1. Introduction

Birth asphyxia is a major cause of neonatal death especially in developing countries and is defined as the inability of the newborn to initiate and sustain adequate respiration after delivery (Ezechukwu, Ugochukwu, Egbuonu, & Chukwuka, 2004). Of the 130 million infants born every year globally, about four million die in the first four weeks of life- the newborn period (Iawn, Cousens, & Zupan, 2005). According to the World Health Organization (WHO), between four and nine million newborns develop birth asphyxia each year. Of these, an estimated 1.2 million die and at least same number develop severe consequences such as cerebral palsy, epilepsy and developmental delay (The World Health Report, 2003). Historically, asphyxia was categorized into two grades of severity namely asphyxia pallida or pale asphyxia and asphyxia livida, indicating the degree of affliction. Infants with asphyxia pallida were generally regarded as severely affected, requiring immediate resuscitation (Haider & Butta, 2006). However, this was replaced by a more objective measure such as Apgar score by Dr Virginia Apgar (1953) an anesthesiologist in 1952.

However using apgar score alone has been found to be very unreliable especially in predicting outcome (Leuthner & Ug, 2004). Controversies have continued even in the 21^St^ century and Papile has commented on apgar score in the 21^st^ century (Papile, 2001). Birth asphyxia remains one of the commonest causes of new born admissions in some centers (Butta 1990; Azam, Khan, & Malik, 2004; Ekwempu, Maine, Olvukobu, Esien, & Kisseka, 1990). Some of the factors that influence the success of management of the illness include; the place of birth, the level of prenatal care, the cause of the birth asphyxia, the gestational age of the neonate, maternal ill health and events during pregnancy and delivery, the availability of resources, including the specialized personnel to manage the disease (Madqbool, 1997). Many of the babies delivered outside the specialized centers for the treatment of these babies in the developing countries do not reach the centers on time as a result of poverty and poor infrastructural development, so that by the time they arrive at these centers, complications have already set in. There are also few of such centers in these countries which are not only under staffed but also are relatively under equipped compared to the developed countries (Nguigi, 2009). Religious beliefs and ignorance also play a major role in the occurrence of birth asphyxia and its complications (Hashima-E-Nasreen, 2009).

The Central Hospital in Warri and the GN children’s and General Medical clinic, one of the four major children’s clinic in Warri, where this review was undertaken both serve about three local Government Areas in Delta State of Nigeria with a combined population of two million three hundred and fifty persons, using the 2006 Nigerian census figures (Federal Republic of Nigeria Official Gazette, 2007).

The review was for a period of eight years, from January 2000 to December 2007. Eight hundred and forty babies with birth asphyxia were managed in these hospitals and the outcome will be discussed. These will include the pattern of presentation, place of birth, the severity of the asphyxia, the possible causes and the outcome of the management.

## 2. Materials and Method

The case notes of all the babies delivered in the hospitals and those neonates referred to the hospitals within the period of review for any ailment including asphyxia neonatorum were retrieved and analyzed. The information obtained included; place of birth, booked or unbooked, gestation age at delivery, mode of delivery, place of prenatal care, events during pregnancy and delivery of the child, socio-economic status of the parents, including the level of education of both parents. Other information obtained include the parity of the mother, the age of the mother, the Apgar score of the baby if recorded, the method of resuscitation if where indicated, the gestational age at birth, the sex of the child and the treatment given before referral, including the time interval between the birth and referral of the child, presence of seizures, cyanosis, pallor, the level of consciousness on admission. Other information obtained included the management instituted at the Central hospital and GN Children and General Medical clinic, and the outcome.

Asphyxia was defined as Apgar score of less than 7 after five munites or baby did not cry immediately after birth (Ibe, 1990). The severity was classified as mild, if the Apgar score at five minutes is 6-7 or required just suctioning to establish a cry (Olowu & Azubuike, 1999). It is moderate if the five minute Apgar score is 4-5, or required stimulation and oxygen administration before a cry and severe if the score is 0-3 and or required major intervention or is associated with seizures central cyanosis or coma, or opisthotonic posture (Ibe, 1990; Olowu & Azubuike, 1999).

Information on the follow up (including subspecialty follow ups) of these patients was documented, including the duration of follow up. Deaths after discharge from the hospitals following birth asphyxia were not included in this study.

## 3. Results

From the twenty six thousand four hundred and twenty thousand neonates delivered or referred to these hospitals, eight hundred and forty had asphyxia, giving a rate of approximately twenty eight per one thousand live birth (28/1000).

The yearly distribution of the incidence of aspyxia neonatorum is shown in table 1. This showed a steady decline from 2005.

**Table 1:** Showing the yearly distribution

Year	Number of Patients
2000	116
2001	107
2002	112
2003	108
2004	109
2005	102
2006	96
2007	90
**Total**	**840**

Five hundred males were seen, compared to three hundred and thirty four females, giving a ratio of 3:2

**Figure 1:**
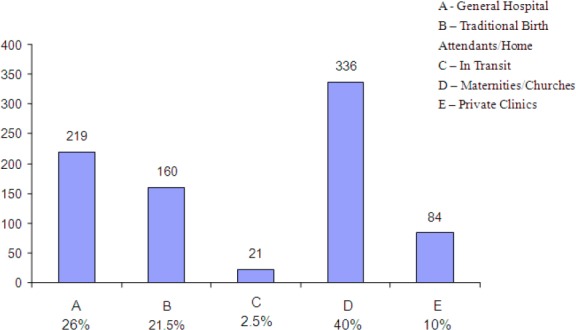
Bar chart showing place of birth

Three hundred and thirty six, representing 40% of the asphyxiated babies were born in maternities and churches. Infact 61.5% of the babies were born in the maternities, churches, traditional birth attendants or at home. About 2.5% or twenty one of the babies were born in transit (delivered on the way to the anticipated place of birth). The mothers of five hundred and four babies did not book for antenatal care representing 60% of the asphyxiated babies.

**Figure 2:**
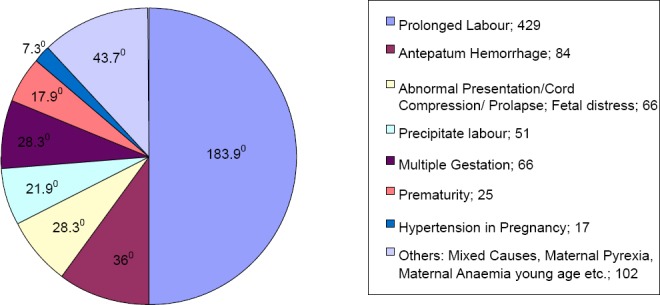
Pie chart showing the causes of birth asphyxia

About 51% of the asphyxiated babies (which is four hundred and twenty nine) resulted from prolonged labour. Antepatum haemorrahage accounted for 10% of the cases. Only 25 of the asphyxiated babies were premature while multiple gestations accounted for 8%. Abnormal presentation including cord prolapse and cord prolapse with fetal distress accounted for 8%. Other causes apart from prematurity include hypertension in pregnancy which accounted for 12% of the cases, age of the mother, maternal pyrexia during the perinatal period, anaemia in pregnancy and others. These are shown in Figure 2.

324 had mild asphyxia which is 38.2% while 250 of these had severe birth asphyxia. These are shown in Table 2.

**Table 2:** Showing the number of babies with various degrees of severities

Degree of Severity	Number	Percentage
Mild	321	38.2%
Moderate	269	32%
Severe	250	29.8%
**Total**	**840**	**100%**

The mortality rate was 27.3% which is two hundred and twenty nine patients. Of these, 52%, about one hundred and nineteen had severe birth asphyxia. 43% of the deceased neonates had moderate asphyxia which translated to ninety eight patients, while twelve of these had mild asphyxia which is 5%. One hundred and fifty three of the deceased children were males and seventy six females, giving a ratio of 2:1.Mortality ratio as shown in table 3 was highest in neonates born in maternity/ churches. The least was from private hospitals with a percentage of 7.5%.

**Table 3:** Showing mortality ratios from various place of delivery

Place of Birth	Number of Deaths	Percentage
Maternity/Churches	125	54.5%
TBA/Home	46	20%
Government Hospital	23	10%
Private Clinics	17	7.5
In Transit	18	8%
**Total**	**229**	**100%**

TBA = Traditional Birth Attendant

## 4. Discussion

Birth asphyxia remains a leading cause of neonatal morbidity and mortality in Nigeria.^1^ A study by Okolo and Omene (1985) in Benin City showed that neonatal mortality dropped from 49/1000 live births to 16.9/1000 from the 1970s to 1981 just by the reduction of birth asphyxia in term neonates (Okolo & Omene, 1985). WHO’s fact sheath (2006) shows that it is a major cause of under- 5 mortality (Mortality Fact Sheath, 2006). A study (2010) in Warri Niger Delta between 2000 and 2007 in the same hospitals showed that the incidence of birth asphyxia was higher than prematurity within the same period (840 for birth asphyxia and 639 for prematurity) (Ugwu, 2010).

The incidence of 28/1000 live births in our study is slightly more than Airede’s experience of 26.5/1000 live births from Fox University, Nigeria (1991) (Airede, 1991) and another study in Nigeria and Central Africa (1993) with incidence of 26/1000 (Kinoti, 1993). It’s much higher than the incidence of 4.6/1000 in Cape town (Hall Dr. Smith, 1996), and a far cry from what obtains in developed countries at less than 0.1/1000 (Badawi et al., 1998). On the other hand, it is much lower than the hospital incidence of 93.7/1000 at Wesley hospital in Ilesha Nigeria (Ogunlesi & Oseni, 2008). Even at the same centers, the incidence varied at different periods. While Okolo and Omene (1985) noted a decrease between the 1970s and the 1980s, Ogunlesi and colleague infact noted an increase when 1994 to 1998 was compared to 1999 and 2003 (93.7/1000 vs. 102/1000 respectively). The marked difference may be in the study design. While Ogunlesi and Oseni (2008) used admitted sick babies as the denominator, we used all newborns seen in our hospitals within the study period. Moreover, criteria for diagnosing asphyxia may vary in both studies. According to the American Academy of Pediatrics and American College of Obstetrics, a neonate is labeled to be asphyxiated if the following conditions are satisfied. (a) umbilical cord arterial pH less than 7 (b) Apgar score of 0-3 for longer than 5minutes, (c) neonatal neurological manifestations such as seizures, coma, hypotonia and (d) multisystem organ dysfunction such as cardiovascular, gastrointestinal, hematological, pulmonary and renal functions (Committee on Fetus and Newborn, 1996); American Academy of Pediatrics and American College of Obstetrics and Gynecology (2002). Following this strict definition will certainly reduce its incidence.

There was also no significant difference in the yearly distribution in Airedi’s study which was carried out over three consecutive years (Airede, 1991). Our study showed a similar experience.

The incidence was higher in males probably because of females resistance to diseases as a result of their XX chromosomes, X being the site of immunoglobulin production, giving them double protection (Susan & Clark, 2009). Birth asphyxia is an index of obstetric care (Haider, Butta, 2006). In general, a study has shown that, 50% of causes of birth asphyxia is Antepatum, 40% intrapatum, with 10% due to postpartum causes (Dilenge, Majnemer, & Shevell, 2001). Our study shows that it was commoner in unbooked than booked patients, which is similar to the experience of Owolabi and collegues (2008) and in Ilesha, and Etuk in Calabar (Etuk, Etuk, Ekoh, & Udoma, 2000).

Our study also showed that the major cause of asphyxia Neonatorum is prolonged labour, which is same result in other center (Shireeni, Nahar, & Mollah, 2009). The incidence and mortality was commoner in babies delivered in maternities and churches and this is the same experience at Wesley hospital in Ilesha (Ogunlesi & Oseni, 2008). The reason is because of ignorance and traditional belief. When a woman is offered a caesarian section due to feto-pelvic disproportion by an obstetrician, she goes to a maternity where she is assured of delivery without operation. Also delivery in churches is believed to offer protection. Feto-pelvic disproportion as in our study is known to cause birth asphyxia (Leclerg, Shretsha, & Christine, 2009). In contrast to the Iesha study however, neonates delivered in private hospital had the least presentation as birth asphyxia (Ogunlesi & Oseni, 2008). This may reflect the level of care given by the pravite practioners at various localities. Our study also showed that mal-presentation is a major cause of this illness. On the whole, while Antepatum problems especially with the fetus are more likely to be the cause in developed countries (McIntosh, 2008), intrapatum problems are likely to be responsible in developing countries (Ellis & de L Costello, 1999; Ellis, Mananadhar, Mahudi, & Costella, 2000). These are majorly preventable, but occur because of lack of manpower, equipments and poor infrastructural development (Ellis, Mananadhar, Mahudi, & Costella, 2000). These are also our experience in this study.

Mortality rate in those with severe birth asphyxia was also similar with other studies (Anochie & Eke, 2005; Hankins & Speer, 2003; Wammanda, Onalo, & Adama, 2007). In fact, WHO in its article “Countdown To 2015: Nigeria: maternal, newborn and child survival indicates that birth asphyxia accounts for 29% of deaths with infections accounting for 26% (Ogunlesi, Ogundeyi, Ogunfoware, & Olowu, 2008).

## 5. Conclusion

Birth asphyxia remains a leading cause of morbidity and mortality in developing countries, not only neonatal mortality but under five mortality. It is also a major setback in achievement of the 4^TH^ millennium goal. This is a major reflection of the obstetric care in the countries. Ignorance and poverty of the populace are other major reasons for birth asphyxia. Prof Babatunde Oshotumihien of Nigera as the minister of health tried to implement a policy where midwives will be posted to rural areas with an incentive of earning triple the salary of their contemporaries in the urban areas (Olusanya & Okolo, 2006). This would have helped to reduce the incidence of birth asphyxia. However a greater impact in the reduction of this preventable illness lies on the provision of basic infrastructures by the government and increase in the budget for health as recommended by World Health Organization (WHO). A propective study of the incidence and complications of birth asphyxia is currently being undertaken in these centres, so as to compare with this study and also obtain true prevalence as some of these patients are lost to follow up.
